# A Novel Instrument for Measuring Older People’s Attitudes Toward Technology (TechPH): Development and Validation

**DOI:** 10.2196/13951

**Published:** 2019-05-23

**Authors:** Peter Anderberg, Shahryar Eivazzadeh, Johan Sanmartin Berglund

**Affiliations:** 1 Department of Health Blekinge Institute of Technology Karlskrona Sweden

**Keywords:** technophilia, aging, internet, health technology, eHealth

## Abstract

**Background:**

The use of health technology by older people is coming increasingly in focus with the demographic changes. Health information technology is generally perceived as an important factor in enabling increased quality of life and reducing the cost of care for this group. Age-appropriate design and facilitation of technology adoption are important to ensure functionality and removal of various barriers to usage. Development of assessment tools and instruments for evaluating older persons’ technology adoption and usage as well as measuring the effects of the interventions are of high priority. Both usability and acceptance of a specific technology or service are important factors in evaluating the impact of a health information technology intervention. Psychometric measures are seldom included in evaluations of health technology. However, basic attitudes and sentiments toward technology (eg, technophilia) could be argued to influence both the level of satisfaction with the technology itself as well as the perception of the health intervention outcome.

**Objective:**

The purpose of this study is to develop a reduced and refined instrument for measuring older people's attitudes and enthusiasm for technology based on relevant existing instruments for measuring technophilia. A requirement of the new instrument is that it should be short and simple to make it usable for evaluation of health technology for older people.

**Methods:**

Initial items for the TechPH questionnaire were drawn from a content analysis of relevant existing technophilia measure instruments. An exploratory factor analysis was conducted in a random selection of persons aged 65 years or older (N=374) on eight initial items. The scale was reduced to six items, and the internal consistency and reliability of the scale were examined. Further validation was made by a confirmatory factor analysis (CFA).

**Results:**

The exploratory factor analysis resulted in two factors. These factors were analyzed and labeled techEnthusiasm and techAnxiety. They demonstrated relatively good internal consistency (Cronbach alpha=.72 and .68, respectively). The factors were confirmed in the CFA and showed good model fit (χ^2^_8_=21.2, χ^2^/*df*=2.65, comparative fit index=0.97, adjusted goodness-of-fit index=0.95, root mean square error of approximation=0.067, standardized root mean square residual=0.036).

**Conclusions:**

The construed TechPH score showed expected relations to external real-world criteria, and the two factors showed interesting internal relations. Different technophilia personality traits distinguish clusters with different behaviors of adaptation as well as usage of new technology. Whether there is an independent association with the TechPH score against outcomes in health technology projects needs to be shown in further studies. The instrument must also be validated in different contexts, such as other countries.

## Introduction

### Background

Older people’s use of technology is increasingly coming in focus with the demographic changes. Gerontechnology (technology for the aging population) is a growing field in transdiciplinary research as well as in the development of new products [1,2]. Previous research into older people’s technology use has identified that the design [3,4] and technology adoption [5,6] perspectives are important to ensure appropriate functionality and remove various barriers for use. Personal factors, such as self-efficacy and proficiency [5,7,8], as well as subjective technology adaptivity [9,10] have also been identified as significant predictors of technology use in old age.

One area that has attracted interest is the use of technology by older adults in various health settings, both in formal health care [11,12] and from salutogenic perspectives [13,14] targeting social isolation and participation. However, there is a strong need to find evidence of the effectiveness and efficiency of health technology interventions, which is becoming increasingly important as the number of available health technology solutions grows [15].

Several instruments for evaluating interactions with health technology exist today. Common theoretical concepts addressed in those instruments are effectiveness, efficiency, hardware/software, perceived ease of use, and satisfaction, although validated psychometric instruments measuring personality traits are sparse [16].

One of the most widely used instruments is the technology acceptance model (TAM) [17] and its subsequent developments [18] for evaluating attitudes predicting intentions to use and how users come to accept and use a particular information technology. In this model, two specific factors determine the user’s acceptance: perceived usefulness and perceived ease of use. Although TAM was not developed specifically for health information technology (HIT), it has found its way into this area as a measurement of end users’ reactions to HIT [19]. Expansions to adapt TAM to a more specific HIT context have been made: the health information technology acceptance model (HITAM) [20]. Application and problematization of TAM toward older persons have also been made: the senior technology acceptance model [21] and HITAM of older persons [22]. The importance of contextual factors [23,24], as well as the usability and acceptability for older adults with mild cognitive impairment and dementia [25] have recently been in focus.

Another widely used model is the Delone and McLean information systems (IS) success model, which considers constructs of intention to use, user satisfaction, and net benefits as the outcomes of three sets of indicators that are information quality, service quality, and system quality [26]. The Delone and McLean IS success model and its extended variants, such as human, organization, and technology-fit [27], have been widely used in health technology [28]. How traits affected by or closely related to technophobia, such as technostress [29], can impact the satisfaction construct in the Delone and McLean IS model has been discussed [30].

Usability is another essential aspect of HIT evaluation. Both the design and evaluation of artifact, efficiency, effectiveness, and satisfaction are important. The Health Information Technology Usability Evaluation Scale [31] and the System Usability Scale [32] are instruments for usability evaluation that have been used in a HIT context [33,34]. In what is an extension of the TAM perspective, Kamin and Lang [10] explored the motivational resources for older persons’ technology use by the concept of subjective personal adaptivity. They found that positive beliefs about the benefits of technology, the time and effort invested to learn how to use technology, and a sense of trustworthiness and safety while using technology is connected to both perceived technology competence and technology usage.

Usability, acceptance, motivation, and adoption of a specific technology or service are important factors in evaluating the impact of a HIT intervention. However, basic attitudes and sentiments toward technology (eg, technophilia) could be argued to influence both the level of satisfaction with the technology itself as well as the perception of the health intervention outcome. This would constitute a personality trait, an underlying factor that would create a preintervention entry level of acceptance and interest, positive or negative. Edison and Geissler [35] argue that this “affinity” toward technology is a more general attitude that precedes the more specific attitudes resulting from the rational (reasoned or planned) process that is measured in TAM. Plociennik et al [36] found that technophilia has a direct influence on perceived usefulness in TAM. Ronit [37] sees technophilia as the enthusiasm toward technology with its rewarded and knowledgeable adoption correlated with both perceived usefulness and perceived ease of use. Kamin and Lang [9,10] showed a correlation between usability and utility and a subjectively perceived interest and competence.

### Technophilia

Technophilia has no universally established definition but generally refers to a strong enthusiasm and love for modern technology. Seebauer et al [38] define it as “an attitude toward ICT [internet and communication technology], representing a subaspect of technology-related values, just as ICT are a subcategory of modern technology.” Osiceanu [39] defines technophilia as an “attraction, enthusiasm of the human individual determined by the activities which involve the use of advanced technologies. It is expressed by easy adaptation to the social changes brought by technological innovations.” Martínez-Córcoles et al [40] suggest that merely enthusiasm and desire is not enough for technophilia, but also an acquired need for (dependency) and joy of having and displaying the latest products/versions (technoreputation). In a working paper, Li and Fuller [41] suggest a definition of technophilia as “positive affective states that arise momentarily in response to an individual’s ICT context that is appraised to exceed his or her expectations and goals.”

In their review of previous related research on technophilia and similar concepts, Seebauer et al [38] found three hierarchically nested perspectives on technophilia. At the top level, they found values connected to general technology-related values reflecting global beliefs in societal progress through technology. At the intermediate level were attitudes referring more specifically to ICT as a part of modern technologies. The lowest level was constituted by a keen interest in and use of a specific technology or service as a subcategory of ICT. Openness toward technology and innovation influences personal dedication to certain technological artifacts and services, whereas feelings of low enthusiasm may work in the opposite direction [38]. Donat et al [42] refer to this lower (negative) end of technology enthusiasm as *technophobia,* and their construct is made up from two opposite ends where technophilia is at the positive end. Nimrod [43] includes this positive end of the spectrum in her construct of a technophobia scale while investigating ICT use among older adults. Osiceanu [39] further views technophobia as the negative feelings about using technology, but also includes perceptions on the adverse effects that technology may have on society in the construct. This is a common way to conceptualize technophobia construct with both factors concerning personal feelings toward technology use together with overall perceptions of technology in society.

In this study, we choose to avoid the complexity of bringing the two concepts of technophilia and technophobia together and simply let technophilia refer to a person’s enthusiasm for and positive feelings toward their technology use and absence of the fears and doubts some older people could have about their ability to manage using new technology. It constitutes a personality trait, an underlying psychological construct [38] that would create a pretechnology and preintervention entry level of acceptance and interest. This could also be connected to other psychological characteristics (ie, the personality traits of openness and neuroticism) [44].

Steinerman et al [45] report that technophilia is a consistent predictor of openness to research participation for older adults and that research with older adults that incorporates technology should consider technophilia to be more successful in recruiting participants for the study.

Older people compared to younger persons are sometimes reported to score lower on technophilia scales [38]. Still, older people are not a homogeneous group, and there may be differences between “younger” old and “older” old individuals. Therefore, it is of importance to create an instrument with the ability to discriminate between high and low technophilia individuals within various groups of older people.

### Objectives

The purpose of this study is to develop a reduced and refined instrument for measuring older people’s attitudes and enthusiasm for technology based on relevant existing instruments for measuring technophilia (named TechPH, short for technophilia).

Technophilia is a general quality for any individual’s relationship to technology that could potentially influence a wide range of aspects of technology use, such as adoption, continuity, and perceived outcome.

In this text, we contextualize this general quality with a focus on older people for use in health technology intervention research as a complement to existing instruments. The new instrument requires that it should be short and simple to make it usable for older people.

## Methods

### Data Collection and Sample

Data were obtained from a sample of participants in the Swedish National Study of Aging and Care (SNAC). SNAC is a longitudinal cohort study of a representative sample of the aging Swedish population that began data collection in 2001. It is a comprehensive, interdisciplinary study that investigates the health and living conditions of the Swedish population aged 60 years and older. A detailed outline of the SNAC study is available by Lagergren et al [46]. Our study sample was based on participants from one of the four regions in the SNAC study, the SNAC Blekinge (SNAC-B) cohort with individuals living in the municipality of Karlskrona.

Data were collected through a questionnaire that was sent out in October 2017 to all participants in the SNAC-B study who were alive in January 2017 (N=878). Of these, 18 had deceased before answering the questionnaire. A total of 659 persons responded, corresponding to a response rate of 77% (659/860). Among nonresponders, 28% (57/201) were unable to respond due to their health conditions (eg, severe dementia or other diseases) and were considered nonusers of ICT. In this study, only individuals who responded that they were ICT users were included (N=374). Demographic data for the study population (ie, age, gender, and educational level) are presented in Table 1.

### Measures and Scale Development

Development of the new short instrument, TechPH, was made in three steps. First, a search was made in Web of Science and Google Scholar for technophilia measurement instruments to use as a background for building the new instrument. To be included, the articles had to contain a psychometric instrument for use on the individual level. Eight relevant instruments were found to match the criteria (Multimedia Appendix 1), of which seven reported a full instrument and were included in this study. Instrument 8 [40] did not include the full instrument in their article and could not be used in the content analysis. Overarching themes (emotional, personal gain, openness/curiosity, competence, general attitudes) were identified through content analysis and items corresponding to these themes were constructed with a five-point Likert scale questionnaire, ranging from 1 (fully disagree) to 5 (fully agree). Following the instruments in the analysis, both questions with a positive and a negative direction were constructed to make sure both the lower and the higher end of the spectrum of technophilia were sufficiently covered. The instruments used in the analysis also gave reason to assume that more than one factor could be present. A total of six to eight questions was considered ideal both for the length of the instrument and for the case that more than one factor would emerge from the factor analysis.

**Table 1:** Demographic data for the study population, including age, gender, and educational level (N=374).

Category		Participants
**Sex, n (%)**		
	Male	196 (52.4)
	Female	178 (47.6)
**Age (years), mean (SD)**		**72.6 (7.1)**
	65-75, n (%)	232 (62.0)
	76-96, n (%)	142 (38.0)
**Education level,^a^ n (%)**		
	Low	94 (26.0)
	Medium	135 (38.0)
	High	131 (36.0)

^a^N=360 for education level. Education was categorized in three groups according to the previous Swedish education system, relevant for the age groups in this study (low: those who did not finish secondary school; medium: those who finished secondary school but no further education; high: those with some form of higher education).

The new instrument resulting from the content analysis was able to build on the previous instruments by including all themes in the same instrument. The analysis showed that none of the seven existing instruments investigated covered all the themes resulting from the analysis, making the new developed short instrument (TechPH) more comprehensive than its predecessors.

Expert knowledge in gerontology from both the medical side (gerontologist) and the technical side (design expert) was used to phrase and formulate the questions for the target group.

Secondly, the questionnaire was pretested and cognitive interviews [47] were made with the target group (eight individuals of both sexes varying in age between 60 and 82 years). The interview persons were given the 8-item questionnaire and were encouraged to think-aloud when they read them. The interviewer would also follow-up with verbal probing (ie, questions about how the interview persons understood the question) based on item wording, terminology, and if the structure was clear and easy to understand. Specifically, the questions “Can you repeat the question I just asked in your own words? Was there anything confusing about this question? What does the word <term> mean to you as it is used in the question? Tell me what you were thinking when I asked about <topic of question>.”

The item questions were then revised according to the feedback from the interviews with respect to the verbal probing. Especially important was to make sure that the questionnaire was using terminology relevant to older people using technology to ensure face validity. The resulting questionnaire items (Table 2) was also pretested on four persons from the target group.

Finally, factor analyses were made. First exploratory factor analysis to see the factor structure and decide on a one or multiple factor solution. Before the exploratory factor analysis, Bartlett test of sphericity was used to ensure significant correlation and Kaiser-Meyer-Olkin test of sampling adequacy for sufficient variance among the items. Maximum likelihood factoring and Promax with Kaiser normalization were used, and only factors with eigenvalues greater than 1 were included.

The reliability of the questionnaire was calculated with the Cronbach alpha coefficient to ensure sufficient internal consistency.

**Table 2:** Descriptive statistics of suggested instrument items (N=374).

Questionnaire item	Mean (SD)	Median
1. I think it’s fun with new technological gadgets	3.40 (1.19)	3.00
2. Using technology makes life easier for me	3.78 (1.27)	4.00
3. I like to acquire the latest models or updates	2.53 (1.34)	2.00
4. I am sometimes afraid of not being able to use the new technical things	2.90 (1.35)	3.00
5. Today, the technological progress is so fast that it’s hard to keep up	3.73 (1.22)	4.00
6. I would have dared to try new technical gadgets to a greater extent if I had had more support and help than I have today	3.10 (1.41)	3.00
7. People who do not have access to the internet have a real disadvantage because of all that they are missing out on	4.13 (1.16)	5.00
8. Too much technology makes society vulnerable	4.10 (1.07)	4.00

Confirmatory factor analysis was then used to verify the factor structure. Measures of fit are reported, such as chi-square statistic and its significance, the adjusted goodness-of-fit Index (AGFI), the comparative fit index (CFI), the root mean square error of approximation (RMSEA), and the standardized root mean square residual (SRMR).

Finally, an index based on sum scores was calculated [48], and sociodemographic attributes and self-assessed technical competence were assessed for group comparisons and as indicators of criterion validity.

## Results

### Exploratory Factor Analysis

The Kaiser-Meyer-Olkin test of sampling adequacy was in the adequate range of 0.76 [49] and the Bartlett test of sphericity (χ^2^_28_=554.1 was significant (*P*<.001), indicating that the items were appropriate for a factor analysis [50].

A two-factor solution for technophilia emerged with an eigenvalue greater than 1 and examination of the scree plot. A one-factor solution was also tested for but showed low internal consistency and was discarded. The two factors were distinctly different with respective clear loadings. The factor with questions regarding positive feelings toward technology (ie, items 1-3) contained items with various aspects of enthusiasm toward technology was accordingly named *techEnthusiasm*. The factor with more negative feelings (items 4-6), contained items with different aspects of anxiety toward technology with respect to use and competency and was named *techAnxiety*.

The questions regarding general attitudes (items 7 and 8) gave low loadings and cross-loaded on both factors above the recommended maximum threshold of 0.32 [51]; therefore, they were removed.

The final six-item solution (Table 3) gave satisfactory loadings of above 0.5 [50], and a total variance explained of 63.5% for the two factors together.

Convergent validity with average variance extracted above 0.5 [50] and discriminant validity shown with only small cross-loadings, together with a factor correlation of −.48. The reliability of the questionnaire, in terms of internal consistency, was calculated by Cronbach alpha (Table 3) with satisfactory results for the small item number (techEnthusiasm Cronbach alpha=.72 and techAnxiety Cronbach alpha=.68).

### Confirmatory Factor Analysis

The final confirmatory factor analysis conducted showed relatively good [52] fit indexes for the two-factor model (χ^2^_8_=21.2, χ^2^/*df*=2.65, CFI=0.97, AGFI=0.95, RMSEA=0.067, SRMR=0.036). The model showed satisfactory (>0.5) standardized factor loadings given the sample size [50] confirming construct validity. Table 4 shows the standardized parameter estimates.

**Table 3:** Exploratory factor analysis loadings and Cronbach alphas.

Item	techEnthusiasm	techAnxiety
1. I think it’s fun with new technological gadgets	0.86	0.01
2. Using technology makes life easier for me	0.62	0.04
3. I like to acquire the latest models or updates	0.60	−0.02
4. I am sometimes afraid of not being able to use the new technical things	−0.07	0.68
5. Today, the technological progress is so fast that it’s hard for me to keep up	0.00	0.75
6. I would have dared to try new technical gadgets to a greater extent if I had had more support and help than I have today	0.09	0.53
Cronbach alpha	.72	.68

**Table 4:** Confirmatory factor analysis standardized factor loadings for TechPH.

Item	techEnthusiasm	techAnxiety
1. I think it’s fun with new technological gadgets	0.88	—^a^
2. Using technology makes life easier for me	0.63	—
3. I like to acquire the latest models or updates	0.61	—
4. I am sometimes afraid of not being able to use the new technical things	—	0.74
5. Today, the technological progress is so fast that it’s hard to keep up	—	0.72
6. I would have dared to try new technical gadgets to a greater extent if I had had more support and help than I have today	—	0.53

^a^Not applicable.

### TechPH Index

A composite score [50] (see Table 5) was created from the six items in the two factors, techEnthusiasm and techAnxiety (the latter reversely coded due to the negative correlation). Each item was weighted with its loading before sum scores were created and averaged [48] and standardized back to a 1 to 5 scale so that the TechPH index could be interpreted on a five-point response scale, ranging from 1 (fully disagree) to 5 (fully agree), where the higher the index indicates a higher level of technophilia.

**Figure 1:**
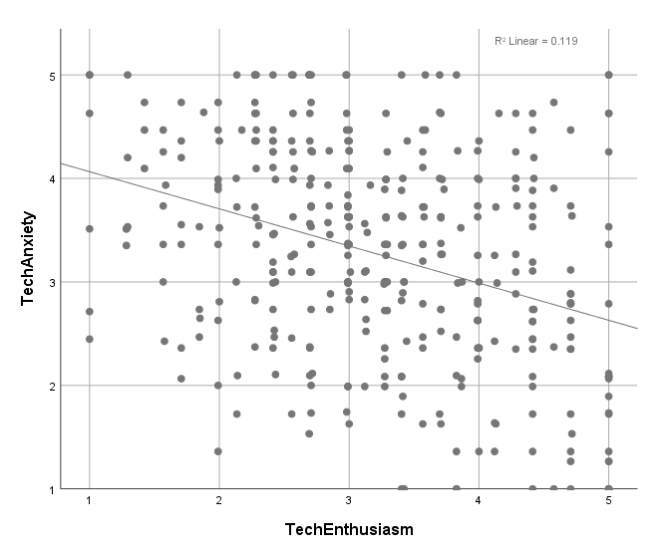
Scatterplot of techEnthusiasm (y-axis) and techAnxiety (x-axis). Individuals showing high technophilia (TechPH) are found in the second quadrant; low TechPH are found in the fourth quadrant.

Distribution of all individuals’ scores in the two factors, techEnthusiasm and techAnxiety, is presented in a scatterplot (see Figure 1). A low negative correlation between the two factors was observed (*R*^2^=.12).

### Group Comparison

The TechPH index (Table 5) was used for group comparison for gender, age group, level of education, self-assessed technical skills, and internet use frequency. This comparison yielded significant (*P*<.05) results. Cronbach alpha was .71, which signifies moderately good reliability of the index and internal consistency.

The TechPH index scores reflect the same finding or assumptions in technology acceptance by demographic variables; the index decreases in the older old (≥75 years) group and is slightly higher for men, which confirms other findings with regard to age groups and gender [53].

**Table 5:** TechPH index: descriptives and group test statistics.

Group		N	TechPH index, mean (SD)	*t* test (*df*)	*F* test (*df*1,*df*2)	*P* value
All		374	3.01 (0.86)	—^a^	—	—
**Gender**				1.71 (372)	—	.046
	Men	196	3.08 (0.88)			
	Women	178	2.93 (0.82)			
**Age (years)**				2.89 (372)	—	.004
	<75	232	3.11 (0.81)			
	≥75	142	2.85 (0.90)			
**Education**				—	0.65 (2,357)	.52
	Low	94	2.96 (0.92)			
	Medium	135	2.96 (0.81)			
	High	131	3.07 (0.87)			
**Self-assessed technical skill^b^**				—	86.40 (2,337)	<.001^c^
	Low	105	2.38 (0.64)			
	Medium	200	3.18 (0.70)			
	High	35	4.02 (0.82)			
**Internet use frequency^d^**				—	29.26 (2,338)	<.001^c^
	Low	77	2.59 (0.82)			
	Medium	104	2.87 (0.67)			
	High	160	3.38 (0.84)			

^a^Not applicable.

^b^“How skilled do you consider yourself when it comes to using a smartphone or a tablet?” Low=not at all skilled, medium=average skilled, high=very skilled.

^c^All post hoc (Tukey) group mean differences were significant at the .05 level.

^d^The participants were categorized as high=daily, medium=at least once a week but not daily, low=less than once a week.

## Discussion

This project set out to create a short instrument to enable measuring of technophilia for use among older persons participating in health technology research projects. The requirements were that it should be based on existing validated instruments and that it should be short and simple to make it usable for older people.

The resulting instrument consists of six items in two factors measuring techEnthusiasm and techAnxiety as factors of technophilia. Factor analysis of the instrument showed the feasibility of a two-factor model. Better fit for a two-factor model, compared to a one-factor model, shows that techEnthusiasm and techAnxiety are not just inverse ends of the same continuum, but two independent factors that could influence various aspects of the use of technology. There is a slight reverse correlation between them (Figure 1).

The results in Table 5 complies with general findings, and assumptions about gender, age, technical skills, and internet use frequency correlates with technology adoption. The correlation between technical skills and TechPH confirms other findings regarding reduced fear of technology by gaining technical skills [54,55]. Seifert and Schelling [56] showed that affinity for technology has a positive impact on internet use. This is also consistent with the findings of Nimrod [43] with similar relations to education, gender, and age, as well as use of technology (in this case, the internet) as in our study. Nimrod investigated technophobia with Sinkovics et al [57] with a 13-item and 3-factor instrument (personal failure, human versus machine ambiguity, perceived convenience). This is a similar setup to TechPH, with different factors pointing both to a lower and higher end of the technophilia/technophobia spectrum. Nimrod measured technophobia specifically toward ICT, such as “computers, internet, and mobile phones,” thus making some of the questions border a measure of a utility perspective rather than a personality trait with feelings toward technology in general.

Concerning techEnthusiasm, item 1 (“I think it’s fun with new technological gadgets”) loaded strongly on the techEnthusiasm factor, which was expected based on the theoretically assumed relationship with the latent variable, confirming that an item (observed variable) that closely reflects the latent variable should be highly correlated with that for a valid model. Item 2 (“Using technology makes life easier for me”) and item 3 (“I like to acquire the latest models or updates”) loaded somewhat weaker but are still seen as conceptually valid for the construct as a whole.

On the techAnxiety side, item 4 (“I am sometimes afraid of not being able to use the new technical things”) closely reflects techAnxiety both in articulation and relatively high correlation. It should be noted that this item considers an internal cause, that is an inability to use technology. Item 5 (“Today, the technological progress is so fast that it’s hard to keep up”) reflects the anxiety over a perceived inability to internalize and relate to the fast, technological progress and connects to a “technostress” [58]. Item 6 (“I would have dared to try new technical gadgets to a greater extent if I had had more support and help than I have today”) refer to the anxiety older people can feel about their lacking ability to handle technology on their own and fear of social isolation and lack of support of the aging population [59].

The two questions regarding general attitudes, items 7 and 8 (ie, “People who do not have access to the internet have a real disadvantage because of all that they are missing out on” and “Too much technology makes society vulnerable”), did not load sufficiently on any of the factors and did not make up a factor of their own. Both these items showed high means and medians and had poor discriminant value, signifying that these attitudes are shared between persons both with high and low techPH. This result is similar to that of Seifert and Schelling [56], in which both onliners and offliners with a high affinity for technology attributed a high value to the internet (in this case, for staying independent longer in old age).

It might be assumed that a high techEnthusiasm score is associated with a low techAnxiety score and vice versa, but it is still possible that both scores could be high or low together. This could have implications for a medium score and needs to be investigated further when TechPH is tested in health technology projects.

An interpretation of a set of high scores in both techEnthusiasm and techAnxiety factors could be that the individual has a basic positive attitude or enthusiasm to technology, but also feels limitations. A lack of interest in technology could be the reason why a person might show low degrees of technology enthusiasm and anxiety simultaneously. However, this lack of interest in technology does not necessarily indicate ignoring technology benefits in general, but it might be the personal attitude about their necessity for the current situation of the respondent [23].

From this perspective, TechPH could be hypothesized to have an effect on the outcome that is separate from the planned intention to use or the perceived usefulness of the application itself. Another assumption is that this could have an impact on how a person perceives problems with use and nonuse friendliness and make a person more error tolerant. This could possibly skew the usability measurements and constitute a confounder to the measured health effect outcome. In smaller studies, especially in randomized controlled studies, this would be a variable of interest to study. This is similar to the effect that Kamin and Lang [10] suggest while exploring the motivational resources for older persons’ technology use by the concept of subjective personal adaptivity. They argue that usability testing might be misleading if motivational factors moderating task performance in person technology transactions are not considered.

Whether this is a personal trait influencing attitudes toward technology that is related to age or physical or cognitive problems will be tested in further studies. We can also assume that the impact on the factors in TechPH is affected differently depending on the type of health technology being evaluated. It could be assumed that the techAnxiety factor has a greater impact on technology that influences items such as personal privacy.

A strength of the instrument introduced in this study is that it is based on previously validated, relevant instruments. It is shortened as much as possible, to three variables per factor [60], and articulated by expert analysis to be suitable for older people. The factor analysis is based on a satisfactory sample size of the general population of older adults from a midsized community in Sweden. Overall, the instrument performed as expected and will now be tested for its prediction ability of the outcome for a health technology project with older people.

In conclusion, we suggest that different technophilia traits distinguish clusters with different behaviors of adaptation as well as usage of new technology and hypothesize that this can be measured with the TechPH score. Whether there is an independent association with the TechPH score or either of the two factors contributing to the score, techEnthusiasm and techAnxiety, against outcomes in health technology projects needs to be shown in further studies. The instrument must also be validated in different contexts, such as other countries.

## Appendix

Multimedia Appendix 1 Content analysis of existing Technophilia instruments.

## Abbreviations

AGFI: adjusted goodness-of-fit index
CFI: comparative fit index
IS: information system
HIT: health information technology
HITAM: health information technology acceptance model
ICT: information and communication technology
RMSEA: root mean square error of approximation
SNAC: Swedish National Study of Aging and Care
SNAC-B: SNAC Blekinge
SRMR: standardized root mean square residual
TAM: technology acceptance model
